# Novel Nuclear Partnering Role of EPS8 With FOXM1 in Regulating Cell Proliferation

**DOI:** 10.3389/fonc.2019.00154

**Published:** 2019-03-19

**Authors:** Adaline Wan Ling Ngan, Michelle Grace Tsui, Danny Hon Fai So, Wai Ying Leung, David W. Chan, Kwok-Ming Yao

**Affiliations:** ^1^School of Biomedical Sciences, The LKS Faculty of Medicine, The University of Hong Kong, Hong Kong, China; ^2^Department of Obstetrics and Gynaecology, The LKS Faculty of Medicine, The University of Hong Kong, Hong Kong, China

**Keywords:** FOXM1, EPS8, nuclear export signal, CCNB1, cell proliferation

## Abstract

One hallmark of cancer cells is sustaining proliferative signaling that leads to uncontrolled cell proliferation. Both the Forkhead box (FOX) M1 transcription factor and the Epidermal Growth Factor (EGF) receptor Pathway Substrate 8 (EPS8) are known to be activated by mitogenic signaling and their levels upregulated in cancer. Well-known to regulate Rac-mediated actin remodeling at the cell cortex, EPS8 carries a nuclear localization signal but its possible nuclear role remains unclear. Here, we demonstrated interaction of FOXM1 with EPS8 in yeast two-hybrid and immunoprecipitation assays. Immunostaining revealed co-localization of the two proteins during G2/M phase of the cell cycle. EPS8 became nuclear localized when CRM1/Exportin 1-dependent nuclear export was inhibited by Leptomycin B, and a functional nuclear export signal could be identified within EPS8 using EGFP-tagging and site-directed mutagenesis. Downregulation of EPS8 using shRNAs suppressed expression of FOXM1 and the FOXM1-target CCNB1, and slowed down G2/M transition in cervical cancer cells. Chromatin immunoprecipitation analysis indicated recruitment of EPS8 to the *CCNB1* and *CDC25B* promoters. Taken together, our findings support a novel partnering role of EPS8 with FOXM1 in the regulation of cancer cell proliferation and provides interesting insight into future design of therapeutic strategy to inhibit cancer cell proliferation.

## Introduction

Sustaining proliferative signaling in cancer cells can result from overproduction of growth factors in an autocrine manner or or constitutive activation of growth factor receptors by mutations (1). The most notable example of growth factor receptor activation is the enhanced expression or activity of various classes of receptor tyrosine kinases (RTKs). Binding of RTKs by growth factors such as Epidermal Growth Factor (EGF) leads to stimulation of their intrinsic intracellular protein-tyrosine kinase activity and the autophosphorylation of multiple tyrosine residues in the cytoplasmic domain of RTKs. The resulting docking sites lead to recruitment and activation of specific SH2-domain-containing signal transduction proteins that can initiate several signal transduction cascades, in particular the Rat Sarcoma (RAS)/Mitogen-Activated Protein Kinase (MAPK) pathway that stimulates cell proliferation. RAS/MAPK signaling affects gene transcription by regulating the activity of transcription factors encoded by immediate early genes and the Forkhead box transcription factor FOXM1 (2). With the expression highly associated with cell proliferation, FOXM1 was found to play roles in the regulation of cell proliferation, apoptosis, metastasis, and DNA damage repair (3–5). Microarray and ChIP-seq analyses have identified important G2/M-specific genes, such as *CCNB1* and *CDC25B*, as direct targets of FOXM1 (6–9), strongly supporting a critical role of FOXM1 in mitosis. To recognize its diagnostic and therapeutic importance, FOXM1 was named Molecule of the Year in 2010.

Interestingly, RTKs can also phosphorylate non-SH2-containing substrates such as the EGF Receptor Pathway Substrate (EPS) 8. EPS8 is constitutively tyrosine-phosphorylated in human cancer cell lines and over-expression of EPS8 was able to transform NIH3T3 cells in focus forming assay (10). EPS8 levels are up-regulated in cancer cells of different tissues of origin [colon, (11); pancreas, (12); pituitary, (13); oral epithelium, (14)] to promote cell migration and invasion via its activation of Rac-dependent actin remodeling. EPS8 contains a N-terminal phosphotyrosine binding protein (PTB) domain, a SH3 domain and a C-terminal “effector region” that directs the sub-cellular localization of EPS8 to filamentous actin (F-actin) in the cell cortex (15). EPS8 forms a ternary complex with ABI1and SOS1 to activate the monomeric Guanosine nucleotide-binding protein (G protein) RAC in actin remodeling [reviewed in (16)]. EPS8 is also known to bind the GTPase-activating protein RN-TRE that modulates the activity of another monomeric G protein RAB5. The RN-TRE-EPS8 complex is believed to inhibit EGFR internalization by promoting the GTP hydrolysis of activated RAB5.

In 2008, Chen et al. (17) provided the first evidence that EPS8 is required for regulation of cancer cell proliferation. Depletion of EPS8 using small interfering RNAs in the cervical cancer cell lines HeLa and SiHa reduced cell proliferation *in vitro* and tumorigenesis *in vivo* when injected into nude mice. Expression of cyclins and p53 were perturbed with an associated change in cell cycle kinetics although the underlying mechanism remains unclear. Wang et al. (18) provided further evidence to support a role of EPS8 in the regulation of squamous cell carcinoma. Over-expression of EPS8 expression in HN4 primary tumor cells increased cell proliferation and migration, and stimulated the expression and promoter activity of *MMP-9*. To explore the regulatory mechanism of EPS8, a microarray screen was performed and *FOXM1* and many of its targets including *MMP-9* were found to be up-regulated (19). Knockdown of FOXM1 expression reduced the proliferation of EPS8-over-expressing cells and EPS8 was shown to enhance *FOXM1* promoter activity (19), suggesting functional crosstalk between EPS8 and FOXM1 but whether they interact directly remains unclear. Recently, EPS8 levels and its sub-cellular localization were found to be tightly regulated during different phases of the cell cycle (20). A transient degradation of EPS8 mediated by SCFFbxw5 is required for proper mitotic progression but how EPS8 may regulate mitosis remains to be explored.

It is worth noting that EPS8 contains a putative nuclear localization signal (NLS) (21), suggesting that the non-SH2 branch of RTK signaling may also affect nuclear function, and EPS8 may interact with downstream components of the SH2 branch of RTK signaling. To isolate FOXM1-interacting proteins, we constructed a bait from amino acids 337 to 437 [corresponding to a highly conserved 100-amino acid domain of FOXM1; (22)] of rat FOXM1 to screen an insulinoma cDNA library (23). Here, we reported the isolation of EPS8 in the screen and subsequent yeast two-hybrid and immunoprecipitation (IP) assays confirmed interaction of FOXM1 with EPS8 as full-length proteins. Colocalization of EPS8 with FOXM1 was found at the G2/M phase and inhibition of the CRM1/Exportin 1-mediated nuclear export enhanced nuclear translocation of EPS8. EGFP tagging and site-directed mutagenesis revealed the presence of a functional nuclear export signal (NES) within EPS8. Consistent with EPS8 playing an important role during cell proliferation, depletion of EPS8 using shRNAs led to slow down of cell proliferation at G2/M phase and suppressed expression of both FOXM1 and its known target CCNB1.

## Materials and Methods

### Yeast Two-Hybrid and IP Analyses

CDNA library construction and screening for FOXM1-interacting proteins using a LexA-based yeast two-hybrid system were described previously (23). The Matchmaker Gold Yeast Two-Hybrid system (Clontech) was employed to confirm the interaction of full-length FOXM1 and EPS8 proteins and to identify the interacting domains using FOXM1 and EPS8 deletion constructs. Yeast two-hybrid assay was carried out according to the manufacturer's instructions [protocol no. PT3024-1 (PR973283)].

IP was conducted according to Ma et al. (2) to detect interaction between endogenously expressed FOXM1 and EPS8. To study association of FOXM1 and EPS8 with the *CCNB1* and *CDC25b* promoters, Chromatin IP was performed as reported in Kwok et al. (24) using antibodies against FOXM1 (C20 from Santa Cruz) and EPS8 (610143 from BD Transduction Laboratories), respectively. *CCNB1* primers: 5′- CGCGATCGCCCTGGAAACGCA-3′ and 5′- CCCAGCAGAAACCAACAGCCGT-3′; *CDC25b* primers: 5′-AAGAGCCCATCAGTTCCGCTTG-3′ and 5′- CCCATTTTACAGACCTGGACGC-3′.

### FOXM1 and EPS8 Vectors and Site-Directed Mutagenesis

Construction of the vectors expressing FOXM1b and FOXM1c have been previously described (2). The expression vector pcDNA3.1/GS-EPS8-V5 was purchased from Invitrogen. For test of protein-protein interaction using the Clontech yeast two-hybrid system, full length and truncated cDNAs of EPS8 and FOXM1 were subcloned into pGBT9 (bait plasmid) and pGAD424 (prey plasmid), respectively. For depletion of EPS8, four EPS8-targeting GIPZ lentiviral shRNAmirs [V2LHS_17662 (#62; 5′-AAATCAATCAGGCTCACAG-3′), V3LHS_17664 (#64; 5′-TTGGAAATCATCCTCAGGG-3′), V2LHS_17665 (#65; 5′-TTGCACATCTCTGTCAATG-3′), V3LHS_314067 (#67; 5′-TGATAAAGATCTTGTTCCA-3′)] and a non-silencing control were purchased from Thermo Scientific. To study the NES within EPS8, site-directed mutagenesis was performed using pEGFP-C2-EPS8-(1-370) as the backbone construct, which was made by PCR-based subcloning into pEGFP-C2 (BD Biosciences). Hydrophobic amino acids within the predicted NES were substituted with alanine residues using the GeneTailor™ Site-Directed Mutagenesis System (Invitrogen). Details of primers for subcloning and site-directed mutagenesis will be furnished upon request. All constructs were confirmed by restriction digestion and DNA sequencing.

### Cell Culture and Transfection

HEK293T, HeLa and C33A cells (obtained from ATCC) were cultured at 37°C under 5% CO2 in DMEM supplemented with 5.9 g/L HEPES, 3.7 g/L Na2CO3, 292.4 mg/L L-glutamine, 100,000 units/L Penicillin G, 80,000 units/L streptomycin sulfate, and 10% (v/v) fetal bovine serum. Transient transfection was performed on cells seeded on coverslips in wells or plates using FuGENE ® HD reagent (Promega) according to the manufacturer's instructions. For suppression of EPS8 expression by RNA interference, cells were transiently transfected with the various shRNA plasmids (shRNAmirs carrying puromycin marker gene) for 72 h. For positive selection of shRNA-expressing cells, transiently transfected cells were subjected to treatment with 0.5 μg/ml puromycin for an additional 72 h before harvesting for immunoblot analysis.

### Cell Cycle Synchronization and Flow Analysis

For immunocytochemical analysis, HeLa cells were synchronized at the G1/S boundary by double thymidine block as previously described (25). Briefly, cells were treated with 2.5 mM thymidine for 17 h, followed by 9 h of release from block and then another 17 h of treatment with 2.5 mM thymidine. Cell cycle distribution was analyzed by propidium iodide (PI) /DNA staining (26) using BD FACSCANTO II cell analyzer at the Faculty Core Facility, HKU. For arresting HeLa at G1 and S phase, cells were treated with 2.5 mM thymidine for 19 h. After the appropriate treatments, cells harvested at various time points were fixed in 70% ethanol. Data were collected using the BD FACS Diva software, and analyzed with ModFit LT 4.0 software.

### Immunoblot and Immunostaining

Immunoblot analysis was conducted as previously described (2). To study the regulation of EPS8 by nuclear export, HeLa cells grown on coverslips were allowed to proliferate to 70–80% confluence under standard culture conditions. Eighteen nM leptomycin B (LMB) (Sigma L2913) was then administered to the culture medium for 4 h before cells were fixed in 4% PFA, subjected to blocking with 3% BSA and then permeabilization with 0.25% Triton X-100 in 1X PBS. For immunostaining, cells were treated with primary antibodies (C20 from Santa Cruz against FOXM1 and 610143 from BD Transduction Laboratories against EPS8) diluted in blocking buffer (3% BSA in 1X PBS) at 4°C overnight, followed by incubation with the fluorochrome-conjugated secondary antibodies using regular procedure. Coverslips were then mounted on microscopic glass slides with Vectashield Mounting Medium (Vector Lab, Inc., Burlingame) with or without DAPI treatment for counterstaining of nuclear DNA. To study the subcellular localization of EGFP-EPS8 fusion proteins, HEK293T cells were transiently transfected with the various wild-type and mutant pEGFP-C2-EPS8-(1-370) constructs using FuGENE HD transfection reagent according to the manufacturer's instructions. Cells were fixed and permeabilized 48 h after transfection. Slides were subjected to fluorescent or confocal microscopy for visualization of the fluorescent signals.

## Results

### Identification of EPS8 as FOXM1-Interacting Protein

**Figure 1:**
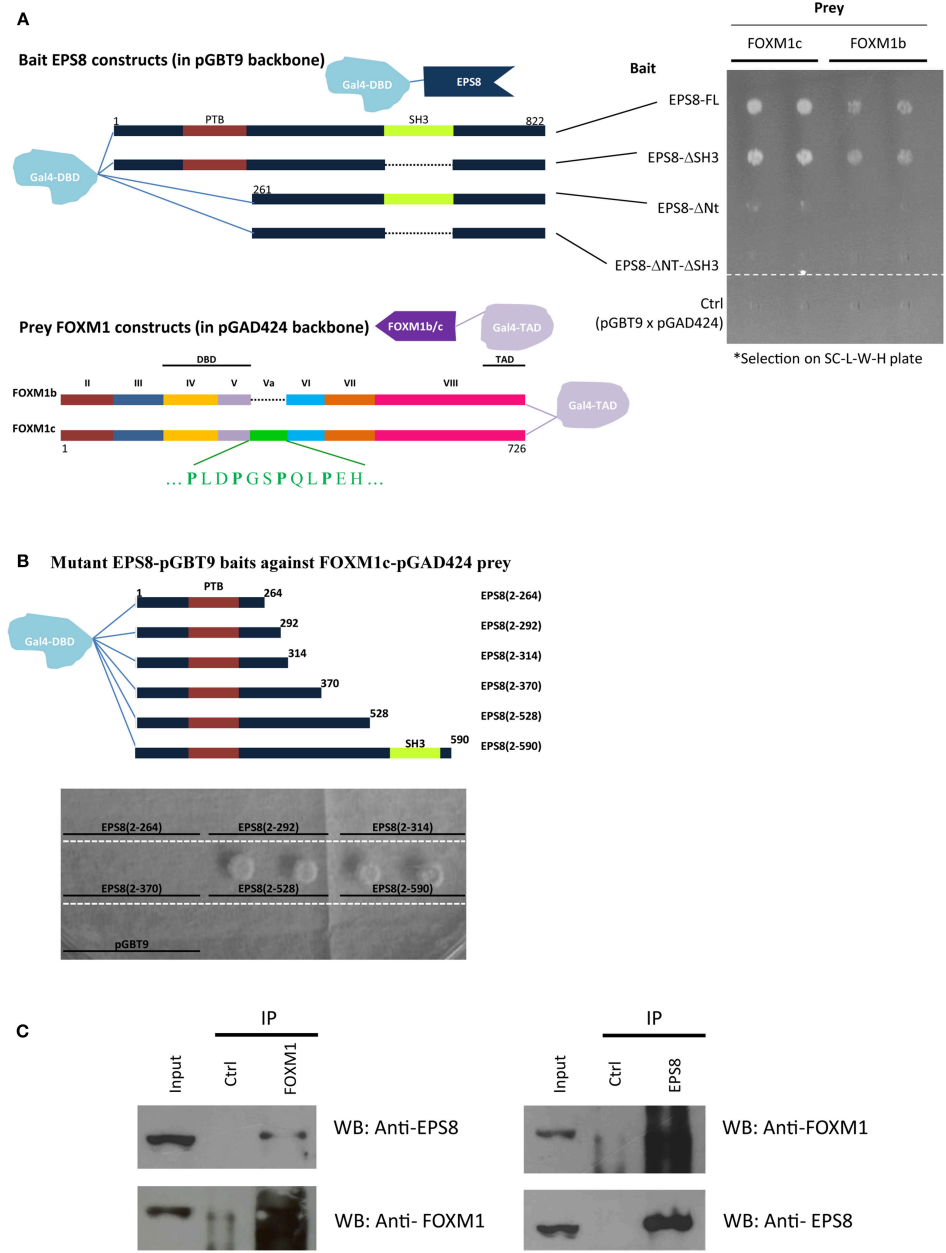
Interaction of EPS8 and FOXM1 in yeast two-hybrid and IP assays. Yeast two-hybrid analysis was performed using the Matchmaker Gal4 Two-hybrid system (Clontech) on SC medium lacking leucine, tryptophan, and histidine (SC-L-W-H). Leucine and tryptophan dropout selected for the presence of the prey and bait plasmids whereas growth in histidine dropout plates indicated the strength of protein-protein interaction. **(A)** Interaction of EPS8 with full-length FOXM1 required the N-terminal region but not the SH3 domain of EPS8. EPS8 interacted with FOXM1c more strongly than FOXM1b. Exon Va (encoding an extra 15 amino acids) is present in FOXM1c but not FOXM1b. Deletion of the SH3 domain (EPS8-ΔSH3) from EPS8 did not affect the interaction but removal of the first 260 amino acids (EPS8-ΔNt), including the PTB domain, led to substantially decreased interaction, suggesting that the N-terminal region of EPS8 contains a critical FOXM1 interacting domain. As negative control, the parental constructs (pGBT9 for bait and pGAD424 for prey) were tested in parallel to rule out background effect. DBD, DNA binding domain; TAD, transcriptional activation domain. **(B)** Test of interaction of deletion constructs extending from the N-terminal region of EPS8 against full-length FOXM1 as prey. The N-terminal domain alone [EPS8(2-264)] was not sufficient for interaction and colony growth occurred when EPS8 extended beyond amino acid 370 in EPS8(2-528). Adding the SH3 domain by extending to amino acid 590 in EPS8(2-590) did not increase colony growth further, consistent with the SH3 domain being dispensable for interaction. **(C)** IP assays. HeLa cell lysates were subjected to IP with anti-FOXM1 antibody and control antibody (rabbit anti-ETS2). Immunoblot analysis using anti-EPS8 antibody indicated that endogenous EPS8 was pulled down. In the reverse IP experiment, FOXM1 could also be immunoprecipitated using anti-EPS8 antibody. Mouse anti-p21 antibody was used as the control antibody.

To gain further insight into the regulatory role of FOXM1, we conducted a yeast two-hybrid screen for FOXM1-interacting proteins using an insulinoma cDNA library (23). To avoid DNA binding and transcriptional activation that might interfere with the LexA-based system, we constructed a bait that contains amino acids 337 to 437 of rat FOXM1. This 100-amino acid region corresponds to the loop region after the DNA binding domain and is the most conserved region within FOXM1 (22). Using this bait, we isolated the EGF receptor pathway substrate EPS8 as an interactor of FOXM1. To confirm this interaction, full-length human EPS8 was constructed as bait using a different yeast two-hybrid system (GAL4-based) to test against full-length human FOXM1 protein of the b or c isoform as prey (Figure 1A). EPS8 interacted with both the b and c isoforms of FOXM1 but the strength of interaction was stronger for FOXM1c, suggesting that exon Va (encoding 15 amino acids, which corresponds to amino acids 337 to 351 of the bait) that is differentially spliced out in FOXM1b contains part of the interaction interface.

To define the interaction domain(s) within EPS8, we made EPS8 constructs deleted of the N-terminus and/or the SH3 domain. Interestingly, deletion of the N-terminal 260 amino acids (which contains the PTB domain; in constructs EPS8-ΔNt and EPS8-ΔNt-ΔSH3), but not the SH3 domain (in construct EPS8-ΔSH3), abolished the interaction. To determine the region within EPS8 that is sufficient to mediate interaction with FOXM1, we constructed EPS8 baits of increasing length starting from the N-terminus. As shown in Figure 1B, the N-terminal ~260 amino acids of EPS8 was necessary but not sufficient for interaction with full-length FOXM1c. Also, EPS8 baits encompassing the N-terminus up to amino acid 370 were not sufficient to interact. Extending EPS8 further to amino acid 528 resulted in strong interaction; the interaction persisted when SH3 was included in construct EPS8(2-590). To test for interaction of endogenous FOXM1 with EPS8 in cervical cancer cells, we employed anti-FOXM1 antibody (C20 from Santa Cruz) to immunoprecipitate FOXM1-interacting proteins from HeLa cell lysate (Figure 1C). Immunoblot analysis detected the presence of EPS8 in the immunoprecipitate. FOXM1 could also be reciprocally immunoprecipitated using anti-EPS8 antibody (610143 from BD Transduction Laboratories). Moreover, the C20 FOXM1 antibody could immunoprecipitate EPS8 in another cervical cancer cell line C33A (Supplementary Figure 1).

### EPS8 Showed Nuclear Localization at M Phase

**Figure 2:**
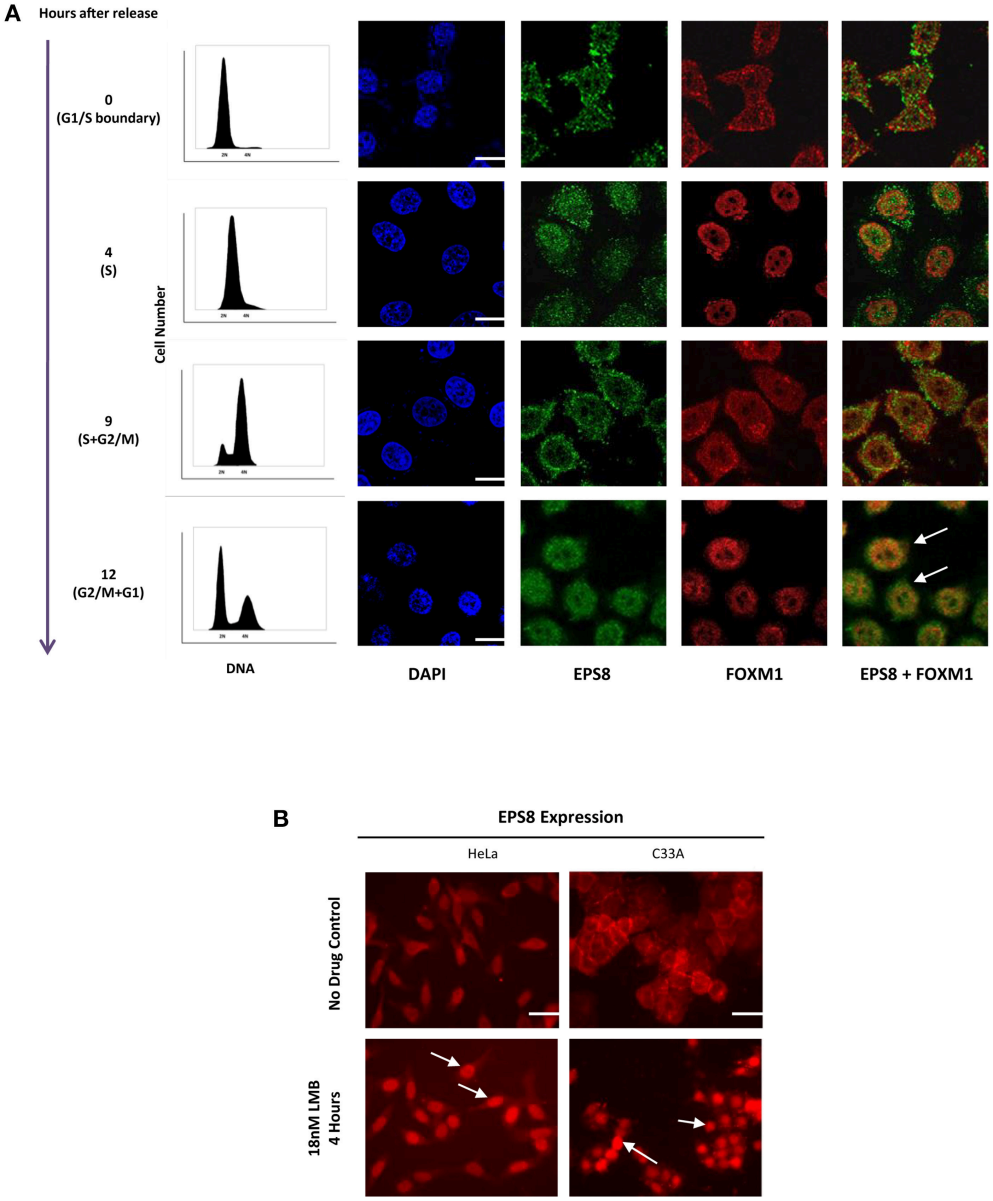
Nuclear entry of EPS8 is cell cycle-phase dependent and is counteracted by nuclear export. **(A)** EPS8 expression in synchronized HeLa cells. HeLa cells were synchronized at the G1/S boundary by double thymidine block. Cells at 0, 4, 9, and 12 h after release from block were fixed for immunostaining to detect the expression of EPS8 (green) and FOXM1 (red). DNA was counterstained with DAPI. Synchronized cells grown in parallel were also subjected to PI/DNA analysis by flow cytometry to study the cell cycle distribution. EPS8 expression was mainly cytoplasmic at 0 and 4 h after release when cells were mostly in G1/S and S phase, respectively. When cells entered into G2 and then M phase of the cell cycle, EPS8 became more perinuclear (9 h after release) and then nuclear (12 h after release). There was significant overlap of EPS8 and FOXM1 expression in the nucleus (white arrows) at 12 h after release. **(B)** Increased nuclear EPS8 expression after Leptomycin B (LMB) treatment. HeLa and C33A cells, with or without treatment with 18 nM of LMB for 4 h, were subjected to immunostaining to detect the subcellular localization of EPS8. Non-treated cells (asynchronized) showed mainly cytoplasmic/perinuclear staining whereas inhibition of CRM1/Exportin 1-dependent nuclear export with LMB triggered significant nuclear localization (indicated by white arrows). Scale bar, 10 μm.

Since FOXM1 is a transcription factor that acts in the nucleus, we studied whether EPS8 showed nuclear localization at the different phases of the cell cycle by synchronizing HeLa cells using double thymidine block. DNA analysis using flow cytometry indicated successful synchronization of HeLa cells at the G1/S boundary (Figure 2A). Synchronized cells progressed to S, G2 and then M phases of the cell cycle at 4 h, 9 h, and 12 h after release from block. Confocal microscopy revealed mostly cytoplasmic and cell surface localization of EPS8 at G1 and S phases. Interestingly, EPS8 assumed a more perinuclear location at 9 h after release followed by its nuclear translocation at G2/M phase (12 h after release), when FOXM1 was also nuclear localized (Figure 2A). To address whether EPS8 is regulated by nucleus export, we treated both HeLa and C33A cervical cancer cells with Leptomycin B (LMB), a specific inhibitor of CRM1/Exportin 1-dependent nuclear export (27). Treatment with 18 nM LMB for 4 h resulted in dramatic re-localization of EPS8 to the nucleus in both cell lines, supporting active exclusion of EPS8 from the nucleus (Figure 2B).

### EPS8 Contained a Functional NES

**Figure 3:**
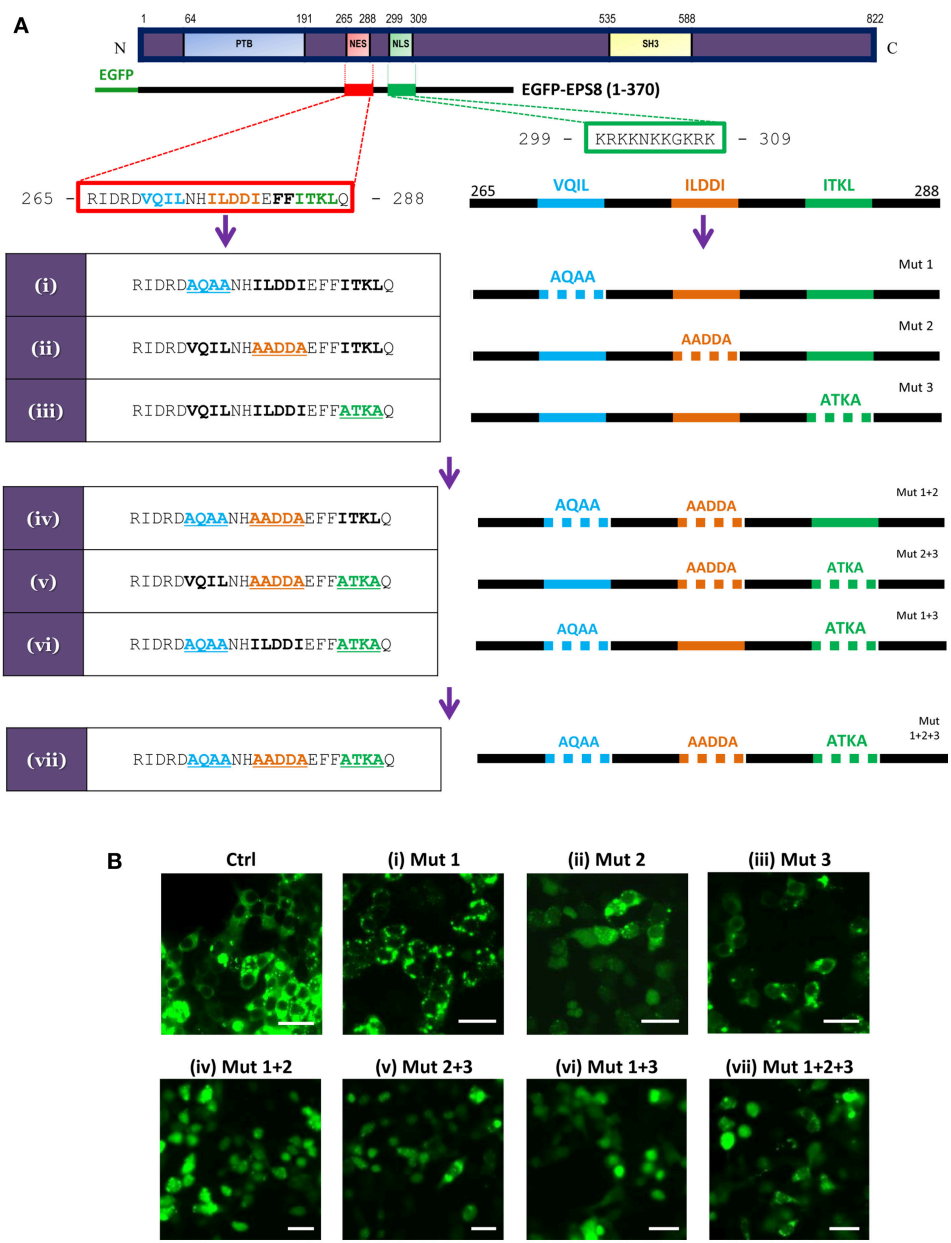
EPS8 contains a functional NES**. (A)** Scheme for systematic alanine-scanning mutagenesis of a putative NES (amino acids 265-288: R**I**DRD**V**Q**IL**NH**IL**DD**I**E**FFI**TK**L**Q; hydrophobic residues in bold) using EGFP-EPS8(1-370) as backbone construct. A putative NLS (amino acids 299-309: KRKKNKKGKRK) is also shown. The three stretches of L/I-rich sequences (VQIL, ILDDI and ITKL) were singly, doubly or triply mutated to generate constructs (i) to (vii). **(B)** Assessing the nuclear export function of the putative NES. Wild-type control (Ctrl) and mutant EGFP-EPS8(1-370) constructs (i) to (vii) were individually transfected into HEK293K cells to assess the extent of nuclear localization of the various EPS8 fusion proteins. Fluorescence microscopy revealed that control (Ctrl) construct and constructs (i) and (iii) showed predominantly cytoplasmic signals whereas construct (ii) directed partial nuclear localization, suggesting that ILDDI is more critical for the nuclear export function. However, all double mutant constructs [(iv), (v) and (vi)] and the triple mutant construct (vii) showed significant nuclear localization, indicating that all three stretches of L/I-rich sequences are required for nuclear export. Scale bar, 10 μm.

CRM1/exportin 1-dependent nuclear export acts via leucine (L)/isoleucine (I)-rich NES in target proteins [Proposed consensus sequences reported as Lx_2,3_(F/I/L/V/M)x_1,2,3_Lx(I/V/L); x = any residue in (28)]. Sequence analysis revealed the presence of a putative NES at amino acids 265 to 288 that partially matches the consensus sequences (Figure 3A). As previously reported in Fazioli et al. (21), a nuclear localization signal (NLS) was predicted at amino acids 299 to 309. This predicted NLS matches the classical monopartite NLS with consensus of B_4_, P(B_3_x), Pxx(B_3_x), B_3_(H/P); B = basic residue (K or R), x = any residue (28). To test whether the predicted NES is functional, we made N-terminal EGFP fusion with the first 370 amino acids of EPS8 for transfection into HEK293T cells. Visualization by fluorescence microscopy indicated nuclear exclusion of the fusion protein (Figure 3B, wild-type control). Three stretches rich in L/I (VQIL, ILDDI and ITKL) could be identified within the putative NES. Three rounds of site-directed mutagenesis were conducted to mutate these three stretches of L/I-rich sequences individually and combinatorially, generating seven mutant constructs [Figure 3A; constructs (i) to (vii)]. As shown in Figure 3B, alanine-scanning mutation of any one of three L/I-rich stretches had little effect on the predominant nuclear exclusion of the fusion protein, with the exception of construct (ii) (Mut 2). However, combinatorial mutations of any two of three stretches or all three stretches led to nuclear import of the fusion protein, suggesting that at least two of the three L/I-rich stretches need to be intact to mediate nuclear exclusion of EPS8. Nuclear localization of the EGFP-EPS8 fusion proteins also hinted that the predicted NLS sequence is functional.

### EPS8 Depletion Suppressed FOXM1 Expression and Delayed Cell Cycle Progression Through G2/M Phase of the Cell Cycle

**Figure 4:**
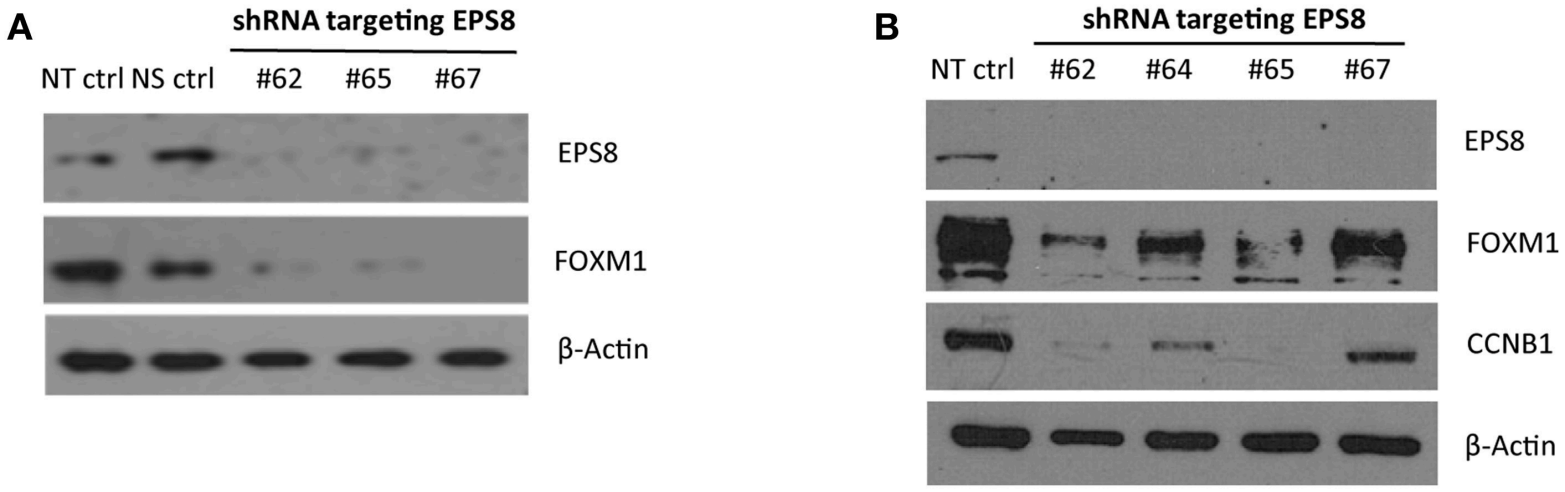
EPS8 Depletion by RNA interference suppresses expression of FOXM1 and CCNB1**. (A)** Transient transfection. HeLa cells transiently transfected with shRNA plasmids targeting EPS8 (#62, #65, and #67) for 48 h were harvested for study of EPS8 and FOXM1 expression by immunoblot analysis. NT ctrl, non-transfected control; NS ctrl, non-silencing shRNA control. β-Actin levels were also studied as loading control. All three shRNA constructs could deplete EPS8 expression and interestingly FOXM1 expression was also suppressed concomitantly. **(B)** Transient transfection followed by puromycin selection. HeLa cells transiently transfected with shRNA plasmids targeting EPS8 (#62, 64, 65, 67) for 72 h were subjected to puromycin (0.5 μg/ml) selection for 72 h. Western blot analysis revealed effective depletion of EPS8. Levels of FOXM1 and the known FOXM1-target CCNB1 were suppressed by all fours shRNA constructs, with the strongest effect observed for #62 and #65.

FOXM1 is critically required for mitosis by regulating *CCNB1* and other M phase target genes, and EPS8 was previously shown to activate *FOXM1* promoter activity. To test whether EPS8, which could interact and co-localize with FOXM1 in the nucleus, modulates expression of *FOXM1* targets and cell cycle progression, we used EPS8-targeting shRNAs to deplete EPS8 in HeLa cells. Compared against the non-specific-targeting shRNA and non-transfected controls, transient transfection of three EPS8-targeting shRNA plasmids (carrying puromycin marker gene) into HeLa resulted in suppression of both EPS8 and FOXM1 expression (Figure 4A). To enrich for transfected cells to effect more complete EPS8 depletion, we also subjected the transfected cells to puromycin selection for 3 days. As shown in Figure 4B, all four shRNAs we tested could suppress EPS8 expression. It is worth noting that #62 and #65 were most effective as reflected by the strongest downregulation of both FOXM1 and the FOXM1 target CCNB1.

**Figure 5:**
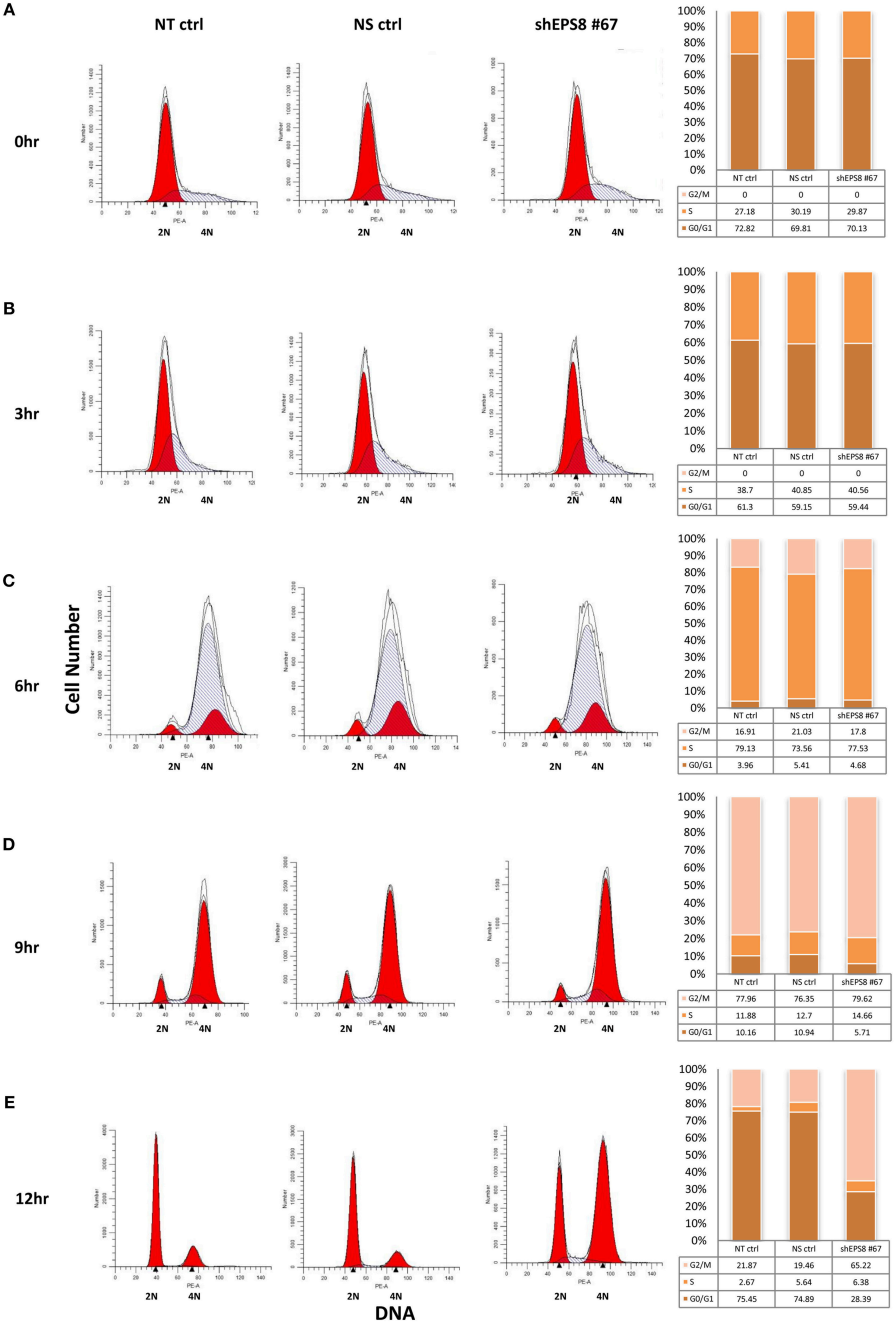
Depletion of EPS8 slows down transition through the G2/M phase of the cell cycle. HeLa cells, transfected with the EPS8-targeting shRNA plasmid #67 (shEPS8 #67) and a non-silencing control shRNA plasmid (NS ctrl) for 42 h, were arrested at G/S and S phase by thymidine treatment for 19 h. Non-transfected cells were also similarly treated as control (NT ctrl). Subsequently, transfected and control cells were released from cell cycle block by replenishment with fresh medium. At the indicated time intervals [**(A)** 0 h, **(B)** 3 h, **(C)** 6 h, **(D)** 9 h, and **(E)** 12 h after release], cells were fixed for PI/DNA analysis of cell cycle distribution by flow cytometry. Because the shEPS8 #67 and NS ctrl plasmids contain a GFP reporter, gating of GFP-expressing cells allowed study of cell cycle distribution of the successfully transfected cells. Percentages of cells at G0/G1, S and G2/M phases of the cell cycle were estimated and presented as bar charts alongside the flow diagrams. At 6 h after release **(C)**, cells previously arrested at G1 and S phase progressed into G2/M phase to similar extent. At 9 h after release **(D)**, the G1 peak started to increase due to G2/M cells reentering another round of the cell cycle. Interestingly, EPS8-depleted cells seemed to lag behind and the difference was most prominent at 12 h after release **(E)**, when the majority (>70%) of NT ctrl and NS ctrl cells reentered G1 phase but only ~25% of the EPS8-depleted cells were at G1. Similar analysis for all the shRNAs tested in Figure 4 was performed but it was only with shEPS8 #67 (which had a weaker knockdown effect to allow cells to stay viable) that we could follow the change in cell cycle kinetics of the shRNA-transfected cells after arrest and drug release. The experiment using shEPS8 #67 was replicated and the same trend of G2/M delay could be observed for EPS8-depleted cells.

To determine how EPS8 deficiency would affect cell cycle progression, we attempted cell cycle analysis for HeLa cells individually transfected with the various EPS8-targeting constructs. However, cells transfected with constructs #62, #64, or #65 exhibited extensive cell death and failed to survive long enough for cell cycle synchronization. Consistent with construct #67 being least effective in suppressing EPS8 expression (see Figure 4B), only cells transfected with this plasmid could be cultured for further cell cycle analysis. We arrested HeLa cells, transfected with EPS8-targeting construct #67, at G1 and S phases by thymidine treatment, followed by monitoring of cell cycle kinetics after release from cell cycle block. Non-transfected cells and cells transfected with the non-silencing shRNA were treated similarly as controls. Presence of the TurboGFP reporter gene in the EPS8-targeting and control plasmids allowed gating of successfully transfected cells for PI/DNA analysis. As shown in Figure 5A, both control and EPS8-depleted cells were arrested at G1 and S phases by thymidine treatment. At 3 h and 6 h after release, cells progressively entered S and then G2 phase of the cell cycle with no discernible difference (Figures 5B,C). At 9 h after release, most cells were at G2/M phase and the G1 peak started to emerge as cells finished mitosis (Figure 5D). Interestingly, EPS8-depleted cells showed a ~50% decrease in the G1 peak when compared to the control cells, suggesting a delayed in G2/M phase. The slowdown in G2/M was most obvious at 12 h after release when most EPS8-depleted cells were still in G2/M phase, whereas the majority of the control cells already returned to G1 phase (Figure 5E).

### EPS8 Was Recruited to the Promoter of *CCNB1* and *CDC25B*

**Figure 6:**
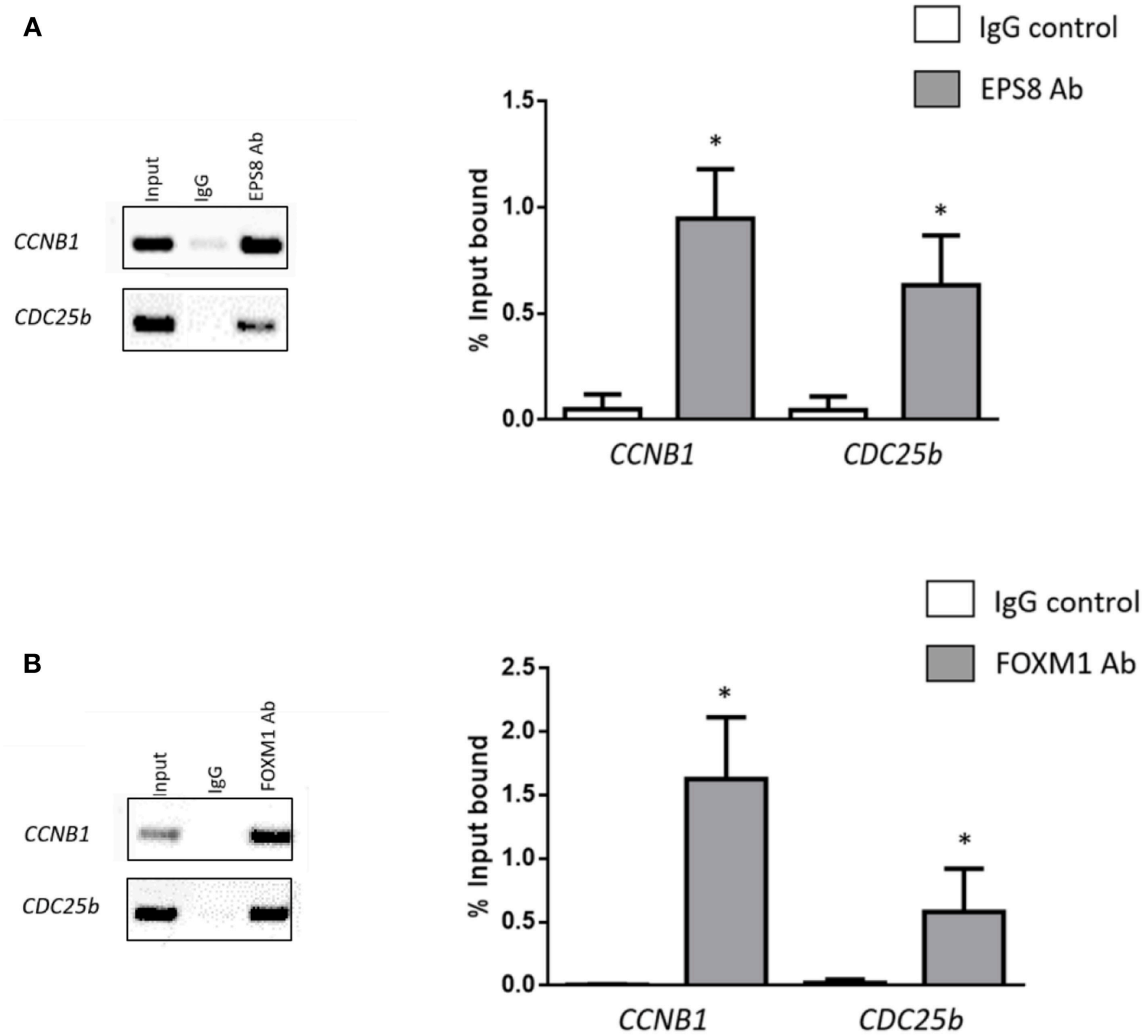
EPS8 shows preferential binding to the promoters of FOXM1 targets. Chromatin immunoprecipitated using EPS8 **(A)** and FOXM1 **(B)** antibodies was subjected to PCR analysis using primers specific to the *CCNB1* and *CDC25b* promoter sequences. Percentage enrichment relative to input as mean ± SEM was analyzed and shown alongside the PCR results. ^*^P < 0.05 when compared with IgG control. *n* = 3.

If EPS8 as FOXM1-interacting protein was directly involved in FOXM1-mediated gene regulation, we would expect chromatin association of EPS8 at the promoter of FOXM1-regulated target genes. To test this notion, chromatin IP (ChIP) was performed using EPS8 and FOXM1 antibodies and the immunoprecipitates analyzed for enrichment of *CCNB1* and *CDC25b* promoter sequences by PCR analysis. As shown in Figure 6, *CCNB1* and *CDC25b* sequences could be pulled down using either EPS8 or FOXM1, but not the control antibody, supporting recruitment of both EPS8 and FOXM1 to the promoter of *CCNB1* and *CDC25b*. Taken together, our findings support that EPS8 is a critical partnering factor of FOXM1 in the regulation of mitotic gene expression and cell cycle progression.

## Discussion

It is well-documented that the proliferation-associated Forkhead box transcription factor FOXM1 is activated by mitogenic signals. Hedgehog signaling upregulates *FOXM1* transcripts transcriptionally (29) whereas RAS/MAPK signaling stimulates the nuclear translocation and transactivating activity of the FOXM1 protein (2). On the other hand, EPS8 is known to enhance the mitogenic effect of EGF signaling (21). Using the most-conserved 100-amino-acid domain, extending beyond the normal carboxyl boundary of the winged helix domain in FOXM1 (22), as bait in yeast two-hybrid screen, we isolated EPS8 as a specific interactor. Full-length FOXM1 was demonstrated to interact with full-length EPS8 in both yeast two-hybrid and IP assays. Immunostaining of synchronized HeLa cells indicated that EPS8 was imported into the nucleus at the G2/M phase of the cell cycle when FOXM1 also reached its peak levels (26). Inhibition of CRM1/Exportin 1-dependent nuclear export by treatment with LMB led to the nuclear import of EPS8 in cervical cancer cells. Importantly, depletion of EPS8 expression in HeLa cells dramatically downregulated the expression of FOXM1 and the FOXM1-target CCNB1, and slowed down transition through the G2/M phase of the cell cycle. Consistent with EPS8 being a partnering factor of FOXM1 in gene regulation, ChIP analysis revealed recruitment of EPS8 to the promoters of both *CCNB1* and *CDC25b*.

The interaction of EPS8 with FOXM1 is likely to be direct as any intermediary factor bridging the interaction would need to be highly conserved and expressed in yeast cells. Previous mass spectrometry analysis of the yeast phosphoproteome indicated that the extent of tyrosine phosphorylation is very low and there are no true protein tyrosine kinases in yeast (30), also arguing against interaction of FOXM1 with phosphorylated forms of EPS8. EPS8 interacted more strongly with the c isoform of FOXM1. Indeed, the most conserved 100-amino acid FOXM1 domain we used as bait contains the 15-amino acid stretch encoded by exon Va, which is specific for the c but not b isoform of FOXM1, at its N-terminus. We believe that exon Va is within the interface of FOXM1 that interacts with EPS8. FOXM1b, present at elevated levels in cancer cells, has been shown to exhibit a greater transforming potential than FOXM1c (31). Whether the higher transforming potential of FOXM1b relates to its weaker interaction with EPS8 remains unclear. EPS8 contains a SH3 domain (amino acids 534-590), which was previously shown to mediate dimerization of EPS8 (32). This EPS8 SH3 domain, being atypical in its binding to the PXXDY consensus but not the canonical XPXXP-containing peptides (33), could be deleted without affecting its interaction with FOXM1. Our deletion analysis revealed that EPS8 required both the N-terminal region (containing PTB domain) and amino acids 371-523 to interact with FOXM1.

Much focus has been put on understanding how EPS8 exerts regulatory effects on cell ruffling and how EPS8 upregulation in cancer cells stimulates cell migration and metastasis (34–36), but its nuclear role remains unclear. Our analysis using EGFP-EPS8 fusion constructs and site-directed mutagenesis indicated that the putative NES and NLS predicted within EPS8 are functional. Interestingly, EPS8 was actively excluded from the nucleus by Exportin 1-mediated nuclear export. Regulated EPS8 nuclear entry was most evident at the G2/M phase of the cell cycle, suggesting that EPS8 is a cooperative factor of FOXM1 in the regulation of mitotic cell division. Indeed, EPS8 downregulation led to a slowdown in transition through the G2/M phase and a concomitant suppression of CCNB1 expression. Moreover, ChIP analysis revealed recruitment of EPS8 to the promoters of *CCNB1* and *CDC25b*. It is worth noting that EPS8 was previously found to stimulate FOXM1 promoter activity (19). Overexpression of EPS8 was shown to stimulate FOXM1 expression transcriptionally using qPCR and transient reporter assays (19). In addition, FOXM1 is known to be subjected to positive autoregulation (37). Our demonstration that EPS8 interacted directly with FOXM1 and that EPS8 downregulation led to suppressed FOXM1 expression suggests that EPS8 might also be recruited to the FOXM1 promoter. We believe that EPS8 functions similarly as cooperative factor in mediating the positive autoregulation of FOXM1. However, we failed to detect recruitment of EPS8 to the FOXM1 promoter by ChIP analysis (data not shown). EPS8 might be recruited far away from the proximal *FOXM1* promoter; further ChIP-seq analysis would be required to sort this out. Most recently, EPS8 was shown to be required for the survival of acute myeloid leukemia (AML) cells both *in vitro* and *in vivo* (38). Although the underlying mechanism remains unclear, a synthetic cell-penetrating peptide derived from the NLS of EPS8 was found to suppress AML cell proliferation and act synergistically with various chemotherapeutic agents. These recent findings also support a critical nuclear function of EPS8 in regulating cell proliferation.

EPS8 depletion was previously shown to slow down cell proliferation in unsynchronized HeLa cells based on cell counting (17). Here, we found that EPS8 downregulation in HeLa cells suppressed both FOXM1 and CCNB1 expression. Monitoring of cell cycle kinetics using synchronized HeLa cells indicated a slowdown at the G2/M phase. This is reminiscent of the aberrant mitotic progression observed in EPS8-dysregulated U2OS cells (20). In their study, EPS8 downregulation at the cell cortex was shown to be essential for cell rounding at M phase. There is concomitant chromatin condensation and chromosomal segregation when cells round up. We believe that the relocation of EPS8 from the cell cortex to the nucleus to mediate its regulatory effect on mitosis (demonstrated in this study) reflects a crosstalk between regulation at the cell surface and gene transcription in the nucleus.

How is the nuclear entry of EPS8 regulated? In a quantitative proteomic study of EPS8 phosphorylation and phosphorylation-dependent protein binding, many tyrosine residues within EPS8 were found to be differentially phosphorylated upon stimulation of receptor tyrosine kinases (39). Of particular interest is pY525 which is most dynamically regulated. This together with two other sites (pY252, pY485) are located within the FOXM1-interacting region defined by our deletion analysis of EPS8. It is interesting to note that pY485 and pY525 were found to bind many proteins involved in nucleocytoplasmic shuttling, including Ran, Importin-5, Exportin-2, Exportin-7, and Transportin-1. It is worth testing whether tyrosine phosphorylation at these sites will affect the regulated nuclear import of EPS8. Recently, Logue et al. (40) demonstrated that the migration stimulating activity of EPS8 is activated by MAPK. Two MAPK phosphorylation sites (S624 and T628) were identified and regulation through these sites were shown to regulate the intracellular redistribution of EPS8. However, it remains unclear whether phosphorylation at these MAPK sites would affect the nuclear import of EPS8.

Taken together, our findings support that FOXM1 (a downstream target of the SH2-dependent branch of RTK signaling) and EPS8 (a non-SH2-containing substrate of RTK signaling) do interact physically and functionally. We argued that EPS8 is a novel partnering factor required for FOXM1 to exert its multiple roles in cancer cell proliferation and migration/invasion, and targeting the FOXM1-EPS8 interaction might provide an alternative anti-cancer strategy.

## Author Contributions

K-MY designed the work, interpreted the data and critically revised the manuscript. AN performed most of the experiments as part of her PhD thesis work. MT and DS also took part in designing and conducting some of the experiments and revised the manuscript. WL made many of the constructs and helped with conducting some of the experiments. DC helped to design the work and advised us on some experiments.

### Conflict of Interest Statement

The authors declare that the research was conducted in the absence of any commercial or financial relationships that could be construed as a potential conflict of interest.

## Supplementary Material

Supplementary Figure 1 Immunoprecipitation assay using C33A cells. Lysates prepared from another cervical cancer cell line C33A were subjected to IP with anti-FOXM1 antibody and control antibody (rabbit anti-ETS2). Immunoblot analysis using anti-EPS8 antibody indicated that endogenous EPS8 was pulled down.
