# Supplementary figures and images for: Novel Nuclear Partnering Role of EPS8 With FOXM1 in Regulating Cell Proliferation
Source: Front Oncol. 2019 Mar 19;9:154. doi: 10.3389/fonc.2019.00154 (PMC6433973; doi:10.3389/fonc.2019.00154)

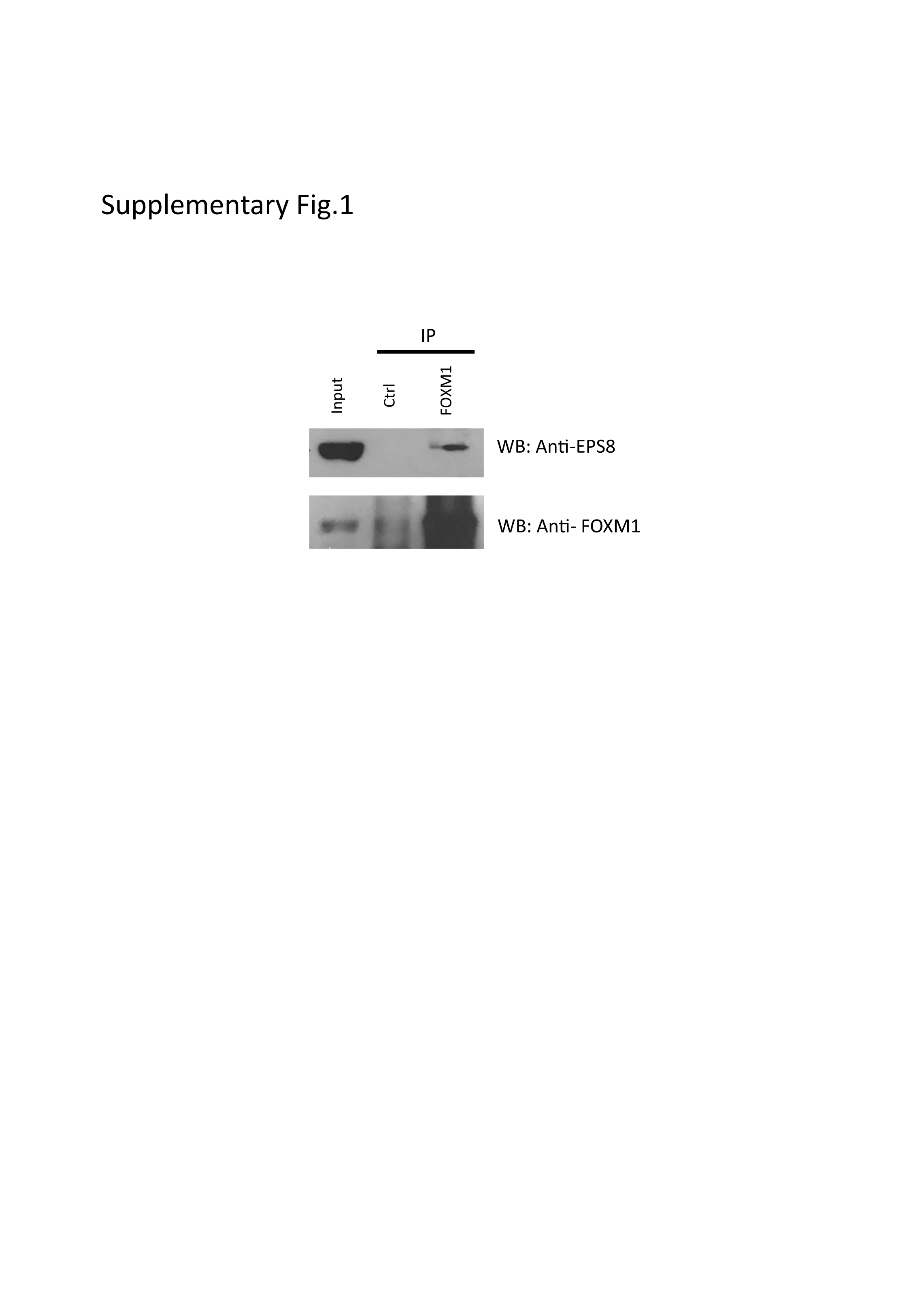

Supplement: Supplementary Figure 1 — Immunoprecipitation assay using C33A cells. Lysates prepared from another cervical cancer cell line C33A were subjected to IP with anti-FOXM1 antibody and control antibody (rabbit anti-ETS2). Immunoblot analysis using anti-EPS8 antibody indicated that endogenous EPS8 was pulled down. [file Image_1.TIF]
